# Inhibition of RSK with the novel small-molecule inhibitor LJI308 overcomes chemoresistance by eliminating cancer stem cells

**DOI:** 10.18632/oncotarget.4135

**Published:** 2015-05-14

**Authors:** Alastair H. Davies, Kristen Reipas, Kaiji Hu, Rachel Berns, Natalie Firmino, Anna L. Stratford, Sandra E. Dunn

**Affiliations:** ^1^ Department of Urological Sciences, Vancouver Prostate Centre, Vancouver, BC, Canada; ^2^ School of Medicine, Queen's University, Kingston, ON, Canada; ^3^ Department of Pediatrics, Child and Family Research Institute, University of British Columbia, Vancouver, BC, Canada; ^4^ Phoenix Molecular Diagnostics, Richmond, BC, Canada

**Keywords:** RSK, YB-1, drug resistance, drug target, cancer stem cells, breast cancer

## Abstract

The triple-negative breast cancer (TNBC) subtype is enriched in cancer stem cells (CSCs) and clinically correlated with the highest rate of recurrence. Several studies implicate the RSK pathway as being pivotal for the growth and proliferation of CSCs, which are postulated to drive tumor relapse. We now address the potential for the newly developed RSK inhibitor LJI308 to target the CSC population and repress TNBC growth and dissemination. Overexpression of the Y-box binding protein-1 (YB-1) oncogene in human mammary epithelial cells (HMECs) drove TNBC tumor formation characterized by a multi-drug resistance phenotype, yet these cells were sensitive to LJI308 in addition to the classic RSK inhibitors BI-D1870 and luteolin. Notably, LJI308 specifically targeted transformed cells as it had little effect on the non-tumorigenic parental HMECs. Loss of cell growth, both in 2D and 3D culture, was attributed to LJI308-induced apoptosis. We discovered CD44+/CD49f+ TNBC cells to be less sensitive to chemotherapy compared to the isogenic CD44-/CD49f- cells. However, inhibition of RSK using LJI308, BI-D1870, or luteolin was sufficient to eradicate the CSC population. We conclude that targeting RSK using specific and potent inhibitors, such as LJI308, delivers the promise of inhibiting the growth of TNBC.

## INTRODUCTION

Despite being identified as particularly aggressive with high rates of relapse and poor survival over a decade ago, treatment and management of triple-negative breast cancer (TNBC) remains a significant clinical problem [1, 2]. Attacking these tumors with anthracycline-based chemotherapies does not improve outcomes [3]. By virtue of the fact that TNBCs are distinguished by the expression of the epidermal growth factor receptor (EGFR) reliance on the MAPK pathway may be their Achilles heel [4], a notion supported by small-molecule screens [5]. Unfortunately, EGFR inhibitors were met with repeated disappointment in clinical trials suggesting that other constituents of the MAPK pathway may be better targets. Large screening efforts using small interfering RNAs identified *RPS6KA3* (encoding RSK2) as being absolutely required to sustain the growth of estrogen-receptor negative breast cancer suggesting it may be a relevant target [6, 7]. Soon following, RSK2 was validated as a target for TNBC *in vitro* and *in vivo* with the unique ability to eliminate the cancer stem cell (CSC) population which is believed to give rise to tumor recurrence [8]. In patients, *RPS6KA3* mRNA is associated with TNBC [9] and poor outcome [8]. In the genesis of TNBC, RSK1 and RSK2 are upregulated as a consequence of expression of the Y-box binding protein-1 (YB-1) oncogene [10]. To date, there have been a small number of pan-RSK inhibitors reported, notably SL0101 [11], BI-D1870 [12], and luteolin [13]. However these small molecules are not highly specific for RSK and therefore more selective small molecules were recently developed, LJI308 and LJH685 [14]. Herein, we evaluated the potential of the new pan-RSK inhibitor LJI308 in inhibiting the growth and survival of TNBC. Further, we addressed its utility in preventing tumor recurrence by eliminating the CSC population, which we show is resistant to most traditional chemotherapies.

## RESULTS

**Figure 1:**
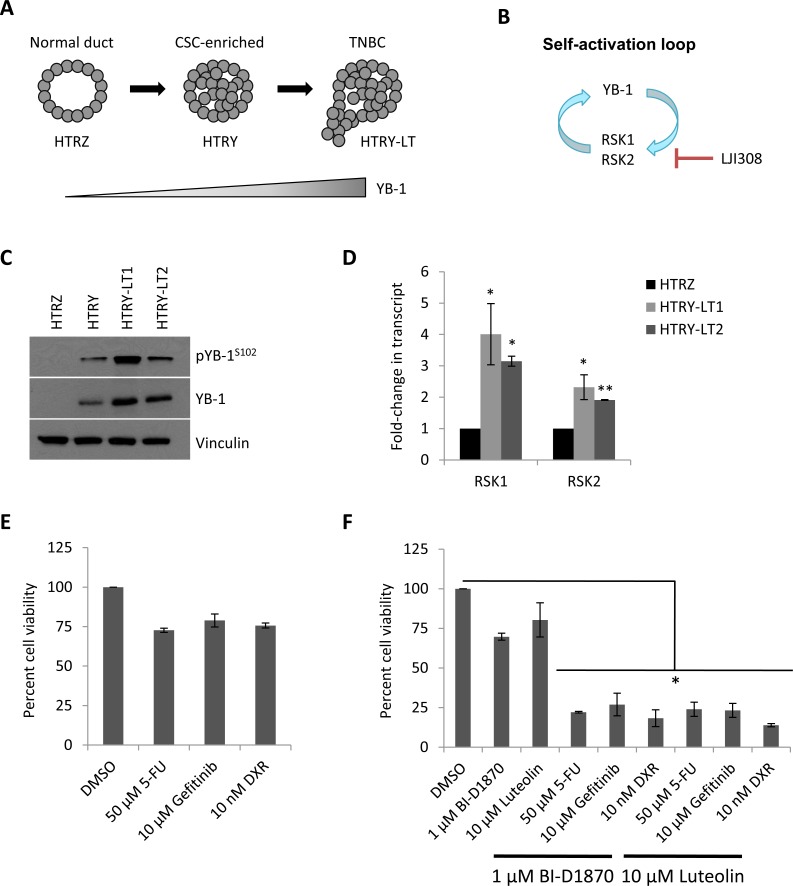
RSK is over-expressed in TNBC and confers multi-drug resistance (**A**) Schematic representation of YB-1-driven breast cancer progression. Long-term YB-1 induction (30 day) transformed HMECs into cancerous cells of the TNBC subtype. (**B**) Schematic diagram depicts a RSK/YB-1 self-reinforcing loop. RSK induces ongoing YB-1 phosphorylation as well as YB-1-dependent RSK expression. (**C**) Immunoblot of YB-1 in HTRZ, HTRY (96-hour induction), and HTRY-LT cell lines. RSK expression had previously been characterized in these lysates (see [10]). Vinculin was used as a loading control in both instances. (**D**) Quantitative RT-PCR of *RPS6KA1* (RSK1) and *RPS6KA3* (RSK2) transcript in HTRY-LT cell lines relative to HTRZ cells. *, *P* < 0.05; **, *P* < 0.01. (**E**) Viability of HTRY-LT1 cells treated with 5-fluorouracil, gefitinib, or doxorubicin for 96 hours was assessed by Cellomics ArrayScan. Data is presented relative to DMSO control. 5-FU, 5-fluorouracil; DXR, doxorubicin. (**F**) Viability of HTRY-LT1 cells treated with BI-D1870 and luteolin alone or in combination with 5-fluorouracil, gefitinib, and doxorubicin for 96 hours. Data is presented relative to DMSO control. *, *P* < 0.05. 5-FU, 5-fluroruracil; DXR, doxorubicin.

Our group recently reported that induction of YB-1 could transform HMECs into carcinoma cells through a CSC-enriched intermediate (Fig. 1A), the phenotypes of which are summarized in Table 1 [10]. This process was driven by a self-activation loop between YB-1 and RSK (Fig. 1B) [10]. The tumorigenicity of these transformed cells was dependent on the level of YB-1 oncogene expression (Fig. 1C) and also correlated with RSK (Fig. 1D). Cultures established following long-term YB-1 over-expression (HTRY-LT1 and HTRY-LT2) formed tumors when transplanted into mice that were molecularly subtyped as TNBC [10]. The sensitivity of these cell lines to three commonly utilized therapeutic agents was measured at their approximate IC_50_ values reported in the established MDA-MB-231 TNBC breast cancer cell line: 50 μM 5-fluorouracil [15], 10 μM gefitinib [16], and 10 nM doxorubicin [17] (Fig. 1E). The HTRY-LT cell lines exhibited broad resistance to these therapies (Fig. 1E). We therefore questioned whether administration of RSK inhibitors could increase the efficacy of conventional chemotherapy. HTRY-LT cells were treated with a sub-lethal dose of RSK inhibitor, 1 μM BI-D1870 or 10 μM luteolin, alone or in combination with 5-fluorouracil, gefitinib, and doxorubicin. A sub-lethal dose of the RSK inhibitors were selected based on the respective IC_50_ values reported in other TNBC models [8, 13]. As expected, treatment with BI-D1870 and luteolin alone had little to no effect on cell viability; however, a synergistic combinatory effect was observed with 5-fluorouracil, gefitinib, and doxorubicin (Fig. 1F). Specifically, cell viability decreased by more than 80% with the combination treatments (Fig. 1F). These data suggest that a RSK inhibitor could be used in combination with traditional chemotherapy to increase drug efficacy.

**Table 1:** Phenotypic characterization of cell lines

	HTRZ (non-tumorigenic)	HTRY (CSC-enriched)	HTRY-LT (TNBC)
Spheroid formation	+	++	++
Invasion	-	+	++
Soft agar growth	-	-	+
Tumor-initiation *in vivo*	-	-	+

**Figure 2:**
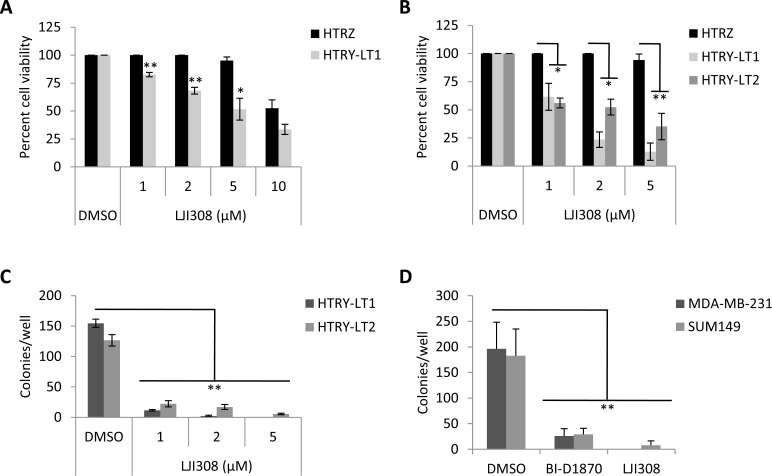
Inhibiting RSK suppresses the growth of TNBC cell lines (**A**) Viability of HTRZ and HTRY-LT1 cell lines treated with increasing doses of LJI308 (1 - 10 μM) for 96 hours. DMSO treated cells served as a control. *, *P* < 0.05; **, *P* < 0.01. (**B**) HTRZ, HTRY-LT1, and HTRY-LT2 cell lines were treated with increasing doses of LJI308 (1 - 5 μM) for 8 days, with repeat dosing at 96 hours. DMSO treated cells served as a control. *, *P* < 0.05; **, *P* < 0.01. (**C**) Soft agar colony formation of HTRY-LT1 and HTRY-LT2 cells grown in the presence of LJI308 or DMSO control. Drug was replenished weekly and colonies quantified at 28 days. **, *P* < 0.01. (**D**) Soft agar colony formation of MDA-MB-231 and SUM149 cells grown in the presence of BI-D1870 (10 μM) or LJI308 (10 μM) assessed after 21 days. **, *P* < 0.01.

**Figure 3:**
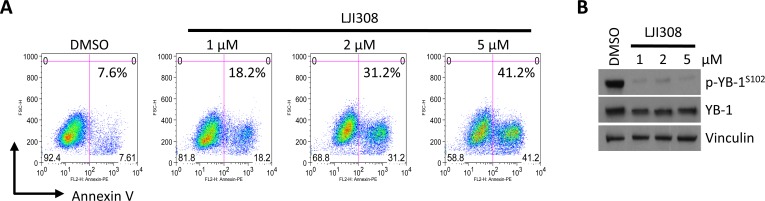
LJI308 kills TNBC correlative with YB-1 inhibition (**A**) Annexin V staining of HTRY-LT1 cells treated with LJI308 analyzed by flow cytometry at 6 days post-treatment. DMSO treated cells served as a control. (**B**) Immunoblot of total and phosphorylated YB-1 in HTRY-LT1 cells treated with increasing dose of LJI308 for 72 hours. Vinculin was used as a loading control.

A hurdle in translating current generation RSK inhibitors into the clinic has been their lack of specificity, for example, BI-D1870 also reportedly targets Aurora B and PLK1 as well as other kinases at high doses [12, 14]. Therefore, there has been a concerted effort to develop highly specific and potent RSK inhibitors, the most recent being LJI308 [14]. Treating HTRY-LT cell lines with increasing doses of LJI308 (1-10 μM) decreased cell viability by up to 90%; however, little to no effect was observed in the non-tumorigenic HTRZ control cells after 4 or 8 days (Fig. 2A, 2B). The cell lines proliferate at a similar rate [10] and thus the decrease in cell growth and viability can be directly attributed to LJI308 treatment. Notably, LJI308 also suppressed the growth of HTRY-LT cells in 3-dimensional soft agar cultures (Fig. 2C). A similar result was observed with the TNBC cell lines MDA-MB-231 and SUM149 (Fig. 2D). The decrease in cell growth was attributed to apoptosis based on an increase in annexin V-positive apoptotic cells following LJI308 treatment (Fig. 3A). These effects were mediated, at least in part, by the ability of LJI308 to inhibit the activation (phosphorylation) of the oncogenic YB-1 transcription factor (Fig. 3B).

**Figure 4:**
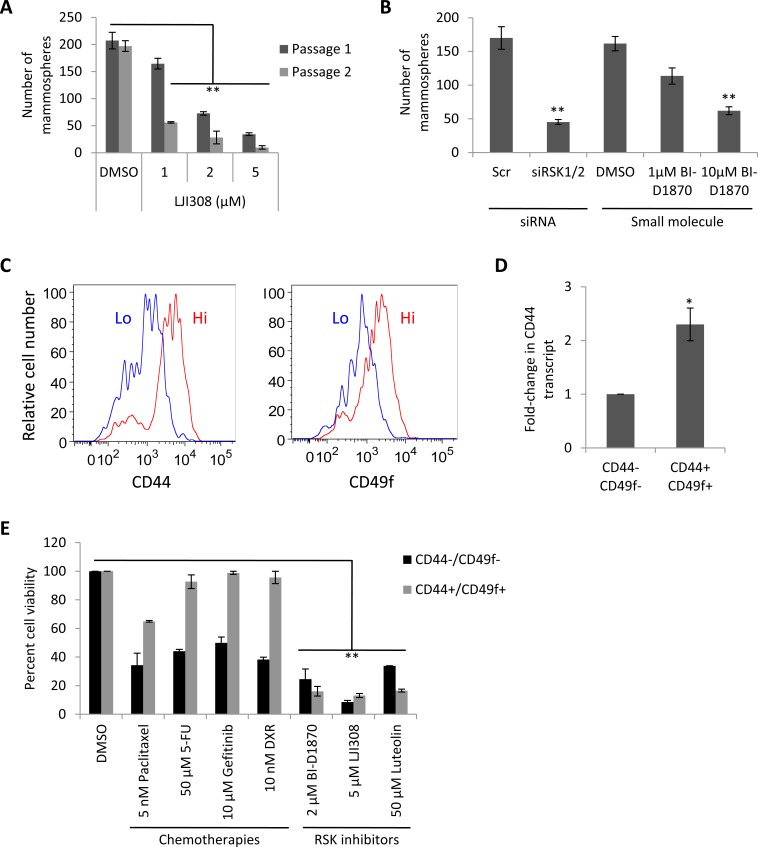
CSCs are sensitive to RSK inhibition (**A**) HTRY-LT1 cells were propagated as mammospheres in the presence of LJI308 (passage 1). After 7 days, the spheroids were dissociated and serially passaged into secondary cultures absent of drug (passage 2). **, *P* < 0.01. (**B**) HTRY-LT1 cells were pre-treated with siRNA targeting RSK1 and RSK2 (20 nM) or the small molecule RSK inhibitor BI-D1870 for 48 hours prior to establishing mammosphere cultures. BI-D1870 pre-treated cells were plated in cultures supplemented with BI-D1870. The number of spheroids was quantified after 7 days. **, *P* < 0.01. Scr, scrambled control. (**C**) Flow cytometric analysis of CD44+/CD49f+ and CD44-/CD49f- populations in HTRY-LT1 cells. (**D**) Quantitative RT-PCR of *CD44* transcript in CD44+/CD49f+ CSC and CD44-/CD49f- cell populations fractioned from the HTRY-LT1 cell line by FACS. *, *P* < 0.05. (**E**) Viability of CD44+/CD49f+ CSC and CD44-/CD49f- populations isolated from the HTRY-LT1 cell line treated with paclitaxel, 5-fluorouracil, gefitinib, doxorubicin, BI-D1870, LJI308, and luteolin for 96 hours. DMSO treated cells served as a control. **, *P* < 0.01. 5-FU, 5-fluorouracil; DXR, doxorubicin.

Due to the proposed role of CSCs in mediating tumor recurrence [18, 19], we wanted to evaluate whether LJI308 could not only eradicate bulk tumor cells but also this highly tumorigenic subpopulation. HTRY-LT cells were grown in spheroid cultures to enrich for CSCs [20], which could be repressed by treatment with LJI308 (Fig. 4A). This effect was mirrored using siRNA against RSK1 and RSK2 or alternatively by exposing cells to BI-D1870 (Fig. 4B). We subsequently isolated the CD44+/CD49f+ CSC population from the HTRY-LT cell line by FACS (Fig. 4C). As expected the CSCs over-expressed CD44 (Fig. 4D) and were found to be largely resistant to paclitaxel, 5-fluorouracil, gefitinib, and doxorubicin chemotherapy relative to the CD44-/CD49f- non-CSC population. We therefore questioned whether CSCs could be eradicated by a lethal IC_90_ dose of RSK inhibitor: 2 μM BI-D1870 [8], 5 μM LJI308, or 50 μM luteolin [13]. We observed a significant decrease in both CSC and non-CSC cell viability, with LJI308 demonstrating the greatest efficacy (Fig. 4E). These data strongly suggest that CSCs are refractory to traditional chemotherapy, but sensitive to RSK inhibitors.

## DISCUSSION

Currently, there are no targeted therapies for TNBC, the most lethal form of breast cancer. While conventional chemotherapy is widely used and initially highly effective, the effect is short-lived; these tumors rapidly re-emerge and disseminate within one year of treatment [21]. Therefore, there is a strong clinical rationale that underlies the search for new targeted therapies. RSK represents a promising targeting point as it phosphorylates numerous downstream substrates associated with cell proliferation and motility [22]. We report the RSK inhibitor LJI308 as one of the first potent and effective targeted therapies for TNBC that, unlike currently utilized drugs, exhibits efficacy in eliminating the CSC population.

CSCs are widely accepted to be strongly therapy resistant and drive tumor dissemination and poor outcome in many cancer types, including breast [23]. Because these cells are enriched in residual breast cancer tumors following chemotherapy they may be responsible for driving relapse [23]. Accordingly, targeting CSCs could represent a promising therapeutic strategy for TNBC, a notion supported by the fact that these tumors have the highest percentage of CD44-positive CSCs compared to the other breast cancer subtypes [24]. Our group has previously reported that TNBC CSCs are dependent on RSK for growth and survival [8]. We have now further validated RSK as a bona fide target in new models of TNBC and identified a specific inhibitor LJI308 that is active in TNBC. Treatment with LJI308 induced apoptosis to eradicate both the CSC and non-CSC populations, suggesting it could be a “silver bullet” that not only debulks the tumor but also targets the “seeds” for cancer regrowth. This is particularly relevant in light of recent studies suggesting that non-CSCs are plastic and can acquire a CSC phenotype [25, 26]. Thus, eradication of existing CSCs may not be sufficient and RSK inhibitors that also target the non-CSCs may be mandatory.

While a major concern in the development of CSC directed therapies is that signaling pathways active in CSCs may also be crucial for normal stem cell survival it has been reported that RSK2 knockout mice develop a normal haematopoietic stem cell compartment [27]. Moreover, our group has shown that treating hematopoietic stem cells with BI-D1870 has little effect at doses toxic to CSCs [8]. Together, this suggests RSK is uniquely linked to the CSC population and, as such, targeting it with inhibitors such as LJI308 has important implications in overcoming drug resistance and treating TNBC.

While our present research has focused on TNBC, RSK is also expressed in other subtypes of breast cancer. In HER2 amplified breast cancer, for example, activation of RSK has been directly linked to trastuzumab resistance [28]. The impact of this study likely transcends breast cancer as RSK has also been linked to drug resistance in other cancer types including prostate cancer [29], melanoma [30], and pediatric medulloblastoma [31].

In conclusion, we propose that the use of RSK inhibitors, such as LJI308, in combination with conventional chemotherapy could be used to overcome drug resistance and improve survival in patients with TNBC. The unique ability of these drugs to target the CSC population might provide sustained remission.

## MATERIALS AND METHODS

### Cell lines and treatments

H16N2 HMECs expressing YB-1 (HTRY) or LacZ (HTRZ) under the control of a tetracycline-inducible promoter were cultured in F-12 media (Gibco, Burlington, Canada) containing 10% fetal bovine serum (FBS), as previously described [10, 32]. MDA-MB-231 (ATCC, Manassas, VA, USA) and SUM149 (Asterand, Detroit, MI, USA) cells were cultured as recommended. Cells were treated with the following drugs: 5-fluorouracil (Sigma-Aldrich, Oakville, Canada), BI-D1870 (Centre for Drug Research and Development, Vancouver, Canada), doxorubicin hydrochloride (Sigma-Aldrich), gefitinib (AstraZeneca, Mississauga, Canada), LJI308 (Novartis Pharma AG, Basel, Switzerland), luteolin (Sigma-Aldrich), and paclitaxel (Sigma-Aldrich).

### Quantitative PCR

RNA was isolated using an RNeasy mini kit (Qiagen, Mississauga, Canada) and reverse transcribed using SuperScript III (Invitrogen, Burlington, Canada). Pre-designed Taqman Gene Expression Assays labelled with a FAM reporter (Applied Biosystems, Streetsville, Canada) were used to detect transcript level.

### Immunoblotting

Proteins were harvested in erythrocyte lysis buffer (ELB) supplemented with protease and phosphatase inhibitors (Roche, Mississauga, Canada), run on a 12% SDS polyacrylamide gel, and transferred to a nitrocellulose membrane. The following antibodies were used: YB-1 (clone EP2708Y; Epitomics, Burlingame, CA, USA), p-YB-1^Ser102^ (Cell Signaling, Danvers, MA, USA), and vinculin (clone V284; Millipore, Etobicoke, Canada).

### Cell viability assay

Cells were plated at a density of 5000 cells/well into 96-well plates. At the endpoint, cells were fixed with 2% paraformaldehyde/PBS and stained with Hoechst 33342 (1 μg/ml; Sigma-Aldrich) for 30 minutes at room temperature. Plate analysis was performed using an ArrayScan VTI high content screening instrument (Thermo Scientific, Waltham, MA, USA). Hoechst 33342-stained nuclei were used as a measure of cellularity.

### Apoptosis analysis

Apoptosis analysis was performed with flow cytometry using the Annexin V:PE Apoptosis Detection Kit (BD Biosciences, Mississauga, Canada). Cells were stained with annexin V (1:20) on ice for 20 minutes prior to analysis.

### Soft agar assay

Cells were embedded in soft agar medium (0.6% agarose) at a density of 5 × 10^3^ per well in a 24-well plate. Drug treatments were replenished weekly. Colonies were quantified at 21-28 days, depending on the cell line.

### Mammosphere assay

Cells were washed through a 40 μm filter to obtain a single-cell suspension and seeded at 2 × 10^4^ per well into ultra-low attachment 6-well culture plates (Corning, Corning, NY, USA) in MammoCult Basal media (StemCell Technologies, Vancouver, Canada). Spheres with a minimum diameter of 50 μm were counted at 7 days. For serial passaging, mammospheres were collected by centrifugation at 350*g* for 2 minutes, dissociated with 0.25% trypsin, and re-seeded.

### Fluorescence activated cell sorting (FACS)

Cells were prepared according to standard protocols and suspended in 2% FBS/PBS on ice before FACS. Cells were sorted on a BD FACSCalibur using PE-conjugated CD44 (clone 515; BD Biosciences) and FITC-conjugated CD49f (clone GoH3; BD Biosciences).
